# Principles of design, supply and usage of clinical laboratory equipment for primary health care in developing countries

**DOI:** 10.1155/S1463924679000596

**Published:** 1979

**Authors:** M. Hjelm, S. S. Brown, F. L. Mitchell

**Affiliations:** 1 Commission of the European Communities Community Bureau of Reference (BCR) rue de la Loi 200 Brussels B-1049 Belgium; 2 Clinical Research Centre Harrow Middlesex UK


### Principles of design, supply and usage of clinical laboratory equipment for primary health care in developing countries*

M.Hjelm,
*Commission of the European Communities, Community Bureau of Reference (BCR), rue de la Loi 200, B-1049 Brussels, Belgium,*
S.S. Brown and F.L. Mitchell,
*Clinical Research Centre, Harrow, Middlesex, UK.*

### Introduction

Automatic chemistry as generally understood in developed countries has little place in the health care of the developing world. The workloads in rural areas do not justify the use of large complex analysers and usually there are not the necessary technological support facilties, suitable power supply or favourable economic and climatic conditions. Equipment is almost entirely designed for, and made in, developed countries, and its application elsewhere is often at best unsatisfactory and at worst disastrous [1,2,3,4]. The main reasons for this situation have been:–

(a) lack of overall goals and relevant specifications for laboratory medicine at various levels of the health care system;
(b) lack of planning and resources for training of personnel, especially within the primary health care system;
(c) insufficient interest from manufacturers in developed countries in producing 'appropriate' equipment at acceptable cost.

The potential demand for simple but accurate equipment suitable for primary health care in developing countries has recently been realised and attempts are now being made to design instruments to cover the specific problems involved. In this work, careful consideration of the special conditions under which the chemical measurements have to be made are required. Particular local conditions are also important. The inclusion of this subject in The Journal of Automatic Chemistry may not at first seem appropriate, but the interpretation of 'automation' must inevitably be modified for different situations. The types of instruments and chemistry systems discussed here can be considered under 'automatic' for the circumstances for which they are intended.

A meeting was convened at the suggestion of the Health Laboratory Technology Office of the World Health Organisation (WHO), Geneva. The object was to review the present situation in the light of the favourable outcome of a pilot project [5] aimed at producing rugged, inexpensive but accurate photometers at the Division of Clinical Chemistry, Clinical Research Centre (CRC), Harrow, England, and their field evaluation, sponsored by WHO [6]. It was attended by representatives of health authorities and manufacturers of laboratory equipment and reagents, together with clinical chemists and haematologists from developing and developed countries.

### Structure of the health care system in developing countries

An attempt to describe in general terms the health care

**Paper based on a World Health Organisation meeting held in Geneva, 10-11 July 1978. A list of participants can be supplied on request to the author.*

**Table 1. Overall structure of the health care system in developing countries and type of personnel responsible for laboratory investigations**

| Level | Type of personnel |
|---|---|
| Primary health care | Primary health worker |
| Dispensary, health centre | Labaratory aide(s) |
| District (rural) hospital (50 beds) | Technician(s) |
| Regional hospital | ( Clinical laboratory scientist(s) |
| Central hospital | ( and senior technician(s) |
| National centre | Advisory experts in clinical laboratory sciences |

**Table 2. Type of laboratory investigations and equipment used at various levels in the primary health care system (including rural fifty-bed hospitals) in developing countries**

| Personnel | Investigation* | Examples of laboratory requirement |
|---|---|---|
| Health worker | U-glucose, qualitative<br>U-Protein, qualitative | Test papers |
| Laboratory aide | *also*<br>B-erythrocytes, inclusion bodies<br>Sp-Microorganisms<br>B-Haemoglobin | <br>Microscope<br><br>Dedicated photometer, pipettes |
| Technicians | *also*<br>S-Albumin<br>S-Total protein<br>S-Calcium<br>S-Sodium<br>S-Potassium<br>S-Iron<br>S-Bilirubin<br>S-Urea<br>S-Glucose<br>S-Enzymes | <br><br><br><br>General purpose photometer.<br>Flame photometer.<br>Balance |

* B=blood, S=serum, Sp=spinal fluid, U=urine

**Table 3. Some specifications for photometers to be used in the primary health care system (including rural fifty-bed hospitals) in developing countries.**

| | Type of simple photometer | |
|---|---|---|
| | **Dedicated** | **All purpose** |
| Use | Pocket size, field and bedside investiations | Laboratory bench investigations |
| Life time | 3-10 years, maintenance free, for B-haemoglobin only | 10 years, repairable |
| Instructions | Basic | Method sheets [11]; trouble shooting manual educational programmes |
| Handling | Precalibrated, non-adjustable | Calibrated with standards; adjustable to concentration readouts |
| Precision | | |
| CV of reading, % | ± 5 | ± 1 |
| of result, % | ± 10 | ± 5 |
| Accuracy % | ± 5 | ± 5 |
| Consumables | Control solutions, cuvettes, diluent, batteries rarely | Standard solutions, reagents, quality control solutions. Cuvettes, batteries |
| Design | Battery-operated, temperature-stable, shockproof, tropicalised | Standard solutions, reagents, quality control solutions. Cuvettes, batteries |

system which typifies developing countries has been made in Table 1. This table also indicates the type of personnel who are responsible for laboratory investigations at the various levels.

It is usual for primary health workers and laboratory aides to be given only a very short period of training, and they therefore have limited skills for performing laboratory investigations. Even so there is a shortage of these workers and it is particularly important that there should be effective training programmes, both for primary health workers and for laboratory aides, in the handling of appropriate laboratory equipment. Careful consideration must be given to assessing the cost effectiveness of providing such equipment and training. The possibility of introducing laboratory equipment at the district (rural) hospital level is heavily dependent on its design and performance characteristics being appropriate for the field situation.

At the higher levels of operation, there are no special differences in skill between practitioners in developing countries and those in developed countries. Often they obtained their basic training in developed countries.

### Need for laboratory equipment at lower levels of the health care system in developing countries

Table 2 lists the laboratory investigations which might cover the most important demands of the primary health care system, including small hospital units with up to fifty beds.

At present, depending on the local situation, few if any of the tabulated investigations may be carried out in the lowest level of health care system. Thus, new equipment should only be introduced if reliable results can be achieved; unreliable results are likely to be worse than no results at all.

An attempt has been made in Table 2 to indicate appropriate laboratory investigations for levels where they might be carried out under favourable conditions, providing that suitable laboratory equipment was available. This should give an indication of the global need for instruments like microscopes and photometers in developing countries.

The basic specifications for the two types of photometers which were the subject of the pilot study [6] at the Clinical Research Centre are given in Table 3. These specifications were drawn up in the light of recommendations put forward by WHO Advisory Groups [7,8].

### The pilot project "Photometers PotLab and MonA" at the UK Clinical Research Centre

At the CRC, a pilot project was started in 1974 to develop simple photometers for use in developing countries. Two types of photometers have been produced, one termed PotLab (Potentiometric Laboratory Colorimeter) intended for use as a dedicated instrument for measuring one blood component, the other, MonA (Monochromatic Absorptiometer) being for general purposes. As is evident from the technical description of the photometers [9], their technical specifications and performance characteristics come close to the specifications listed in Table 3.

During the development of PotLab and MonA, several versions of the instruments were tested in a number of developing countries, mostly under the auspices of WHO [6]. The experience gained from this field work has been used to include a number of new technical features in the final versions. It is hoped that these innovations will contribute towards supplying developing countries with adequate equipment. It is also evident that PotLab and MonA will have a place in the primary health care in many developed countries, in veterinary medicine and blood transfusion services [10].

### Introduction of the PotLab and MonA concept in developing countries

### Production of the instruments

In general, only low cost equipment would be used in the primary health care system of developing countries. The possibility of producing instruments for this use will depend on the following factors:

(a) large volume. High cost technology is usually necessary to produce low cost equipment. Trade restrictions often make it difficult to produce equipment in developing countries. Collaboration with industry is required to find means of overcoming the current financial and logistic deterrents to manufacturing specifically for developing countries.

(b) efficient transport and distribution systems. Transport costs and an efficient local distribution system will increase the selling price of a low cost product to several times the manufacturer's cost.

(c) encouragement from governments and willingness among economically responsible authorities to allocate the necessary funds.

For the instruments to be effective in health care in developing countries, the *user's* costs for PotLab and MonA should not exceed US \$50 and \$ 250 respectively.

### Introduction of the instruments

The effective introduction of equipment for primary health care in developing countries would require:–

(a) systematic training programmes for the use of equipment in a limited number of clinically urgent investigations;

(b) development of reliable analytical procedures taking into account the need for reagents suitable for the local situation [11].

(c) an efficient support service for equipment and reagents.

### Design, supply and use of other types of laboratory equipment

In order to reach defined goals, equipment other than photometers, such as simple pipettes and microscopes must also be introduced. It might even be necessary to develop a type of mobile laboratory especially designed for use in developing countries, in which all basic laboratory tests could be carried out. This need not be more complex than a simple bench supplied complete with all necessary equipment.

The experience gathered during the development of the PotLab and MonA concepts should be useful for this type of project.

### General conditions

There was general agreement that the PotLab and MonA concepts fulfilled most specifications for photometers to be used at lower levels in the primary health care of developing countries.

It was recommended that WHO should take an active part in the implementation of PotLab and MonA concepts by:

(a) supplying information on the suitability of equipment for developing countries especially to organisations concerned with such supplies, e.g. UNICEF;

(b) supporting the development of suitable analytical procedures;

(c) involving international professional organisations, e.g. International Federation of Clinical Chemistry (IFCC), International Committee for Standardization in Haematology (ICSH), and the International Union of Immunological Societies (IUIS), in the development of methods and training programmes.

(d) seeking involvement of the health care industry in both the development aspects of projects and in overcoming the problems that have so far limited availability of equipment designed specifically for developing countries.

It was recommended that WHO should take steps to produce specifications for a general clinical laboratory system, dedicated to the lower levels of primary health care in developing countries, because of the expected importance such a system would have for the screening and treatment of common disorders.

All participants agreed that the meeting had been a valuable forum for the exchange of ideas between interested parties and similar meetings should be organised on a continuing basis.

ACKNOWLEDGMENTS

During the development of this project, many people world-wide, too numerous to mention, have helped with advice, hospitality, evaluation work, etc. Their collaboration is gratefully acknowledged.

REFERENCES

[1] Clinical chemistry for developing countries. Report of the joint International Federation of Clinical Chemistry and World Health Organization Meeting, September 20-24 1971, Geneva, Switzerland.

[2] Mitchell, F.L. The indigenisation of pathology. *Annals of Clinical Biochemistry,* 1975, **12**, 45.

[3] Mitchell, F.L. Simplified instrumentation for the clinical laboratory. *Bulletin of Pan American Health Organisation,* 1976, **10**, 212.

[4] Bull, G.M., Mitchell, F.L. The provision of clinical laboratory services in developing countries. *Tropical Doctor,* 1978, 8, 104.

[5] Brown, S.S. A British approach to technology for rural care: 'appropriate' clinical laboratory systems. CS10 Communications (1979), in press.

[6] Rideout, J.M. *The Journal of Automatic Chemistry,* 1979, 1,(4), 223.

[7] WHO Consultation on Standardization in Haematology, Bilthoven, April/May, 1975. Document LAB/75.3.

[8] WHO Consultation on Standardization in Clinical Chemistry, Geneva, February 1975. Document LAB/75.2.

[9] Pocock, S.N., Rideout, J.M. *The Journal of Automatic Chemistry*, 1979, 1(4) 222.

[10] Hjelm, M, The use of 'push the button' analysers in clinical chemistry. Organisation des Laboratoires-Biologie Prospective. III$^{e}$ Collogue de Pont-a-Mousson, 1975. L'expansion Scientifique Francaise, p.11.

[11] Wilding, P. and Kennedy, J.H. Manual of Routine Methods in Clinical Chemistry for use in intermediate laboratories. 1978, WHO LAB/78.1.

### A total systems approach to laboratory automation

**Peter B. Stockwell**

*Laboratory of The Government Chemist, Stamford Street, London SE1 9NQ, U.K.*

The concept of laboratory automation is a subject of much confusion. One of the major reasons for this is that there is no common understanding of the terminology itself as a subject except in a clinical sense. For example in the latest edition of Kirk-Othmer: Encyclopedia of Chemical Technology [1] no direct reference to automated analysis is made; it includes only a reference to biomedical automatic analysers, thus neglecting a wide area of the use of automated analyses. The International Union of Pure and Applied Chemical (IUPAC) definition of automation clearly excludes most systems commonly marketed as automatic. It states "automation is the use of combinations of mechanical devices to replace, refine, extend or supplement human effort and facilities in the performance of a given process, in which at least one major operation is controlled, without human intervention, by a feedback system". Almost all developments described as automatic are classified within the IUPAC definition of mechanisation; this states "mechanisation is the use of mechanical devices to replace, refine, extend or supplement human effort". A more simple definition of automation which is widely accepted by workers in the field is, "the use of any facility either electronic or mechanical, which eliminates some aspect of manual interaction and improves the efficiency of the analytical process". In this context, feedback mechanisms have little positive advantage to offer at present, although the use of microprocessor techniques clearly modifies this situation. According to the definition which has been adopted for this paper, automation in laboratory analysis can be applied at any of the various stages throughout a procedure and attempts to minimise the level of routine operator intervention. In addition, it attempts to improve the level of quality control by the use of mechanical, electronic and computer techniques in a cost effective manner. Automatic analysis is further confused, deliberately by commercial suppliers on occasions, by the automation aspects of an instrument purporting to be fully automatic but referring to only part of the analytical procedure; most often, this is the measurement stage. A

